# Real-world data on tolerability of COVID-19 vaccination in patients with rheumatoid arthritis based on patient-reported outcomes

**DOI:** 10.1093/rap/rkae111

**Published:** 2024-09-05

**Authors:** Martin Feuchtenberger, Magdolna Szilvia Kovacs, Anna Eder, Axel Nigg, Giovanni Almanzar, Martina Prelog, Arne Schäfer

**Affiliations:** Medizinische Klinik und Poliklinik II, University Hospital Würzburg, Würzburg, Germany; Rheumatologie, MVZ MED BAYERN OST, Burghausen, Germany; Rheumatologie, MVZ MED BAYERN OST, Burghausen, Germany; Rheumatologie, MVZ MED BAYERN OST, Burghausen, Germany; Rheumatologie, MVZ MED BAYERN OST, Burghausen, Germany; Department of Pediatrics, University Hospital Würzburg, Würzburg, Germany; Department of Pediatrics, University Hospital Würzburg, Würzburg, Germany; Medizinische Klinik und Poliklinik II, University Hospital Würzburg, Würzburg, Germany; Psychodiabetologie, Diabetes Zentrum Mergentheim, Bad Mergentheim, Germany

**Keywords:** arthritis, rheumatoid, SARS-CoV-2, COVID-19, vaccination, safety, tolerability, patient-reported outcomes (PROs)

## Abstract

**Objectives:**

To assess tolerability of COVID-19 vaccination in patients with RA and controls based on patient-reported outcomes (PROs).

**Methods:**

In total, 266 study participants were included at 6 ± 1 weeks after their second vaccination (BioNTech/Pfizer (72.2%), AstraZeneca (18.8%) and Moderna (9.0%)). In a cross-sectional, observational study design, PRO data were recorded regarding both total and symptom-level tolerability.

**Results:**

Overall tolerability was very high according to the patients’ self-assessment scores (1.71 for the first and 1.72 for the second vaccination, 6-point Likert scale [1 (very good) to 6 (very poor)]) and did not differ significantly between patients with RA (*n* = 204) and controls (*n* = 62). Self-rated overall tolerability regarding first vaccination was significantly better (*P* = 0.002) in patients receiving mRNA vaccines (*n* = 193, mean tolerability 1.59) as compared with vector-vaccinated patients (*n* = 73, mean tolerability 2.04). Homologous or heterologous vaccination regimens had no statistically significant effect on vaccine tolerability (*P* = 0.131). Reservations about the vaccination were rare (6.4% for the first and 6.0% for the second vaccination) but significantly associated with poorer overall tolerability (*P* < 0.001) and significantly reduced willingness to recommend vaccination to others (*P* < 0.001 for the first and *P* = 0.004 for the second vaccination).

**Conclusion:**

Based on these real-world data, tolerability of COVID-19 vaccination was very good in both RA patients and controls. Reservations against COVID-19 vaccination were rare overall, but if present, associated with a significantly worse tolerability and a significantly lower degree of recommendation.

Key messages

- COVID-19 vaccination was very well tolerated from the patients’ perspective based on patient-reported outcomes
- High level of recommendation of the vaccination to others among RA patients and controls
- Concerns about vaccination were significantly associated with worse reported tolerability and lower rates of recommendation to others

## Introduction

According to John Hopkins University, the number of deaths during the COVID-19 pandemic worldwide to date amounts to more than 6 million [1]. Based on a recently published mathematical modelling study, vaccination against COVID-19 has prevented nearly 20 million deaths from COVID-19 worldwide [2]. Considering these findings, among others, there is no doubt in the scientific community about vaccination against COVID-19 being a crucial measure in the fight against the SARS-CoV-2 pandemic. This is of particular relevance for patients with a congenital or acquired immune impairment, such as those with immune-mediated inflammatory diseases (IMIDs) and DMARDs [3]. As patients with IMIDs were excluded from the pivotal trials, data on the immunogenicity and tolerability of COVID-19 vaccines in patients with IMIDs are lacking from the original vaccine trials leading to vaccination approval [4, 5]. However, the efficacy of vaccination has now been increasingly well studied also in patients with IMIDs: although there are indeed differences in immunogenicity depending on age, comorbidities and the immunomodulatory therapy used, it is also considered effective in these patients. Scientific associations such as the ACR and the EULAR therefore gave recommendations for vaccination in patients with IMIDs [6, 7]. Despite all these achievements, there are still concerns, particularly on the part of patients, about the safety of COVID-19 vaccination [8–12]. In addition to the immediate tolerability of the vaccination, there are also concerns about the potential to trigger flares of the underlying rheumatic disease [13]. In the case of SARS-CoV-2, vaccine scepticism may have been further exacerbated by the novel vaccines, e.g. those based on mRNA technology. Potentially resulting reservations are likely to have a considerable impact on the extent to which patients comply with a vaccination recommendation. Therefore, a robust database on the safety and tolerability of COVID-19 vaccination is essential for physicians to inform and advise patients in their daily routine. The present study focuses on tolerability aspects of a double vaccination against COVID-19 in patients with RA compared with controls. We hypothesized that the overall self-reported tolerability of the COVID-19 vaccination would be good and comparable between included subgroups (RA patients and healthy controls). This cross-sectional, observational study is based on a structured interview of patients 6 ± 1 weeks after the second vaccination against COVID-19 to assess tolerability in a qualitative and quantitative approach and thus exclusively reflects the patient’s point of view.

## Materials and methods

### Patient recruitment

In this cross-sectional, observational study, we included a total of 266 individuals with RA (*n* = 204) and 62 immunologically healthy patients. All study participants were consecutively recruited 6 ± 1 weeks after their second vaccination against COVID-19 with either a homologous or heterologous vaccination scheme with COVID-19 vaccines (BioNTech/Pfizer; AstraZeneca; Moderna). A heterologous vaccine regimen was defined as the use of different types of COVID-19 vaccines for the first and second doses. Eligible patients were included and examined in the rheumatological outpatient clinic of MVZ MED BAYERN OST, Burghausen, Germany, within an observational single-center and cross-sectional study design. All participants were recruited between 12 April 2021 and 14 September 2021. RA patients were treated with DMARDs and received MTX, Janus kinase (JAK) inhibitors, TNF inhibitors or rituximab (RTX), whereas control patients were diagnosed with osteoarthritis of the hands and not under immunomodulatory medication. Inclusion criteria for the RA subgroup comprised a confirmed diagnosis of RA according to ACR-EULAR 2010 criteria, an age of at least 18 years, as well as written informed consent to study participation.

Exclusion criteria were a relative or absolute contraindication for therapy with the abovementioned DMARDs, a previously known intolerance of anti-rheumatic drugs, and a history of PCR-confirmed SARS-CoV-2 infection since beginning of the pandemic. The main sociodemographic characteristics of the total study sample (stratified by patient subgroups with and without RA) are displayed in Table 1. Moreover, we investigated the association between the presence of RA or DMARD therapy with different patterns of tolerability.

**Table 1.** Patient characteristics and relevant medical data (by patient subgroups with and without RA and DMARD therapy

	RA patients with DMARD therapy	Reference group without RA	*P* value	Mean difference (95% CI)
	(*n* = 204)	(*n* = 62)		
Age (years)	67.2	64.3	0.050	2.9 (0.0, 5.7)
Female sex (%)	66.7	79.0		
Male sex (%)	33.3	21.0	0.064	n.a.
Mean RA disease duration (years)	11.3	0	n.a.	n.a.
Seropositivity (%)	83.3	0	n.a.	n.a.
Prednisolone use (%)	18.1	0	n.a.	n.a.
Mean dose prednisolone (mg/day)	4.3	0	n.a.	n.a.
Diabetes mellitus (%)	15.7	12.9	0.516	n.a.
Mean GFR values (ml/min)	75.5	81.3	0.032	5.8 (0.5, 11.0)
Mean RR syst. (mm Hg)	143.5	143.0	0.947	0.5 (−14.4, 15.4)
Mean RR diast. (mm Hg)	83.4	83.4	0.986	0.1 (−8.5, 8.6)
Overall tolerability (first vacc.)	1.7	1.9	0.328	0.2 (−0.1, 0.5)
Overall tolerability (sec. vacc.)	1.7	1.8	0.396	0.1 (−0.2, 0.4)
BioNtech (%)	73.5	67.7		
AstraZeneca (%)	17.6	22.6		
Moderna (%)	8.9	9.7	0.646	n.a.
SARS-CoV-2 IgG (BAU/ml)	282.6	345.1	<0.001	62.5 (33.7, 91.2)

*P* values shown are not corrected for multiple comparisons. Seropositivity was defined as positivity for RF and/or ACPA [21, 22].

Dependent variables or outcome variables were so-called PROs, meaning that the status of a patient’s health condition (here: symptoms associated with [in-]tolerability of the vaccination) came directly from the patient. This means there was no interpretation of the patient’s response by a clinician or anyone else. 6 ± 1weeks after administration of the second vaccine dose, defined symptoms or symptom domains were queried using a self-constructed questionnaire (Supplementary Data S1, available at *Rheumatology Advances in Practice* online). In addition to specific physical symptoms (dichotomous recording: present *vs* not present), patients were asked about their impression about general tolerability of the vaccination (ordinal data level—6-point Likert scale according to e.g. school grades/academic grading scale in Germany [1 (very good) to 6 (very poor)]). Further PROs referred to the patients’ attitudes towards vaccination: ‘Were there any reservations beforehand?’ and ‘Would the patient recommend the vaccination to others afterwards?’.

All study participants provided written informed agreement for participating in the study and for the publication of the scientific data retrieved. The study was approved by the ethics committee of the University Hospital Würzburg, Würzburg, Germany (207/21-me). The organization and realization of the study were in accordance with the principles of ‘Good Clinical Practice’ and the Declaration of Helsinki 1964 and its later amendments [14, 15].

Inclusion criteria for the RA subgroup comprised a confirmed diagnosis of RA according to ACR-EULAR 2010 criteria, an age of at least 18 years, as well as written informed consent to study participation. Exclusion criteria were a relative or absolute contraindication for therapy with the abovementioned DMARDs, a previously known intolerance of anti-rheumatic drugs and a history of SARS-CoV-2 infection. The reference group consisted of individuals without RA or any kind of IMID. Therefore, these study participants were without any immunomodulatory medication.

### Measurements of tolerability

Our primary endpoint in this study was to measure the subjective tolerability of the COVID-19 vaccination as evaluated by PROs. Data were captured using a self-constructed questionnaire completed by study participants. As mentioned above, symptoms referring to putative vaccination side effects were coded dichotomously: local pain, swelling at injection site, hardening at injection site, nausea, vomiting, loss of appetite, feeling sick, diarrhoea (more than three loose stools a day), headache, fatigue, myalgia defined as unspecific muscle pain, arthralgia defined as unspecific joint pain, fever (body temperature ≥38.5°C measured by skin thermometer) and chills. Self-assessment of overall tolerability as the primary outcome variable was recorded using ordinal data level.

### Statistical analysis

Presented sample size calculations relate to the primary study analysis (comparison of self-assessed total vaccination tolerability in patients with RA and controls). The sample size calculation assumed two independent samples, a 5% significance level and 95% statistical power to detect a medium effect size (*d* = 0.5). The optimal sample size for two-sided testing was calculated to be at least 220 subjects. For these calculations, we used the software G*Power (version 3.1.9.6).

Data management and statistical analyses were realized using Microsoft Excel (version 16.53) or SPSS (German version 17.0.0) software, where appropriate [16]. All inferential tests were considered statistically significant at *P* < 0.05. We did not perform alpha adjustment for multiple comparisons as our primary focus was on the exploratory nature of the analyses, aiming to identify potential trends and associations with respect to self-reported vaccination tolerability.

Pearson chi-square tests were used to compare frequencies of categorical variables between patient subgroups. Moreover, for the primary outcome measure (overall vaccination tolerability), we used Mann–Whitney *U* tests to test for differences in central tendency between subgroups with and without RA. Pearson correlation coefficients were used to determine bivariate linear associations between dependent variables. For the simultaneous inclusion of multiple predictors, an ordinal regression analysis was performed. For the purely descriptive illustration of central tendencies when comparing self-assessed tolerability of vaccination among patient subgroups, the means (and standard deviations as well as 95% confidence intervals where applicable) were used.

## Results

Investigating the demographic baseline data, most remarkable group differences were the higher age of RA patients (as compared with controls; *P* = 0.050), the higher percentage of male patients (*P* = 0.064) as well as well as the significantly lower glomerular filtration rate (GFR) values (*P* = 0.032). Among the 204 patients with RA, administered DMARDs were distributed as follows: 80 patients received MTX (30.1%), 51 patients were treated with JAK inhibitors (19.2%), 45 patients with TNF inhibitors (16.9%) and 28 individuals with RTX (10.5%). Both, mRNA and vector vaccines were used in the total sample: 73 patients were initially vaccinated with the AstraZeneca vector vaccine (27.4%), 177 participants were vaccinated with the BioNtech mRNA vaccine (66.5%) and 16 patients were vaccinated with the Moderna mRNA vaccine (6%). This distribution changed slightly in favour of BioNtech’s vaccine with the second vaccination: Fifty patients received a second dose with AstraZeneca vaccine (18.8%), 192 patients with BioNtech (72.2%) and 24 patients with Moderna vaccine (9.0%). A total of 243 patients received a homologous vaccination regimen (91.4%).

A very important and remarkable finding was that the overall tolerability in the total study sample (*N* = 266) was high according to the patients’ self-assessment scores on the 6-point Likert scale: This self-rated overall tolerability of the vaccination was excellent, namely 1.71 for the first and 1.72 for the second vaccination.

**Figure 1.**
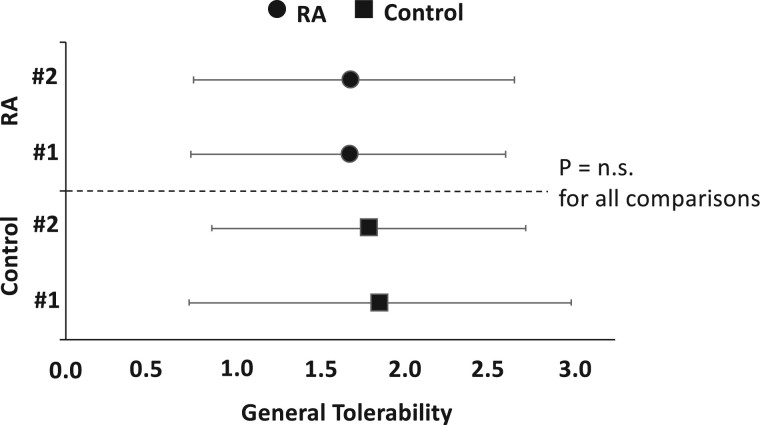
General tolerability by patient subgroup (RA *vs* controls) and by vaccination time point (#1 *vs* #2). Dependent variable (general tolerability; here: *x*-axis) represents a six-point Likert scale (lower = better tolerability). Central tendency is represented as group mean (s.d.). *P* = n.s. for all between-group comparisons

In this context, we were interested in investigating to what extent the presence of RA was associated with the tolerability of the vaccination. However, there was no statistically significant difference between the control group and the group of RA patients, neither in terms of overall tolerability nor in terms of symptom-level tolerability: For the first vaccination, respective mean values regarding general tolerability were 1.85 *vs* 1.67 (controls *vs* RA patients; *P* = 0.328). For the second vaccination, we observed similar data: 1.79 *vs* 1.70 (controls *vs* RA patients, *P* = 0.396; see Fig. 1).

Additionally, we assessed the immune response through SARS-CoV-2 antibody titres, which were significantly different in both subgroups (*P* < 0.001; see Table 1 and Supplementary Fig. S1, available at *Rheumatology Advances in Practice* online). However, our findings revealed no statistically significant difference in tolerability with respect to vaccination response in both RA patients and control subjects: There was no clinically relevant correlation between tolerability and the antibody response in RA patients (r = 0.076; *P* = 0.279) or in controls (r = 0.048; *P* = 0.712) (Supplementary Fig. S2, available at *Rheumatology Advances in Practice* online).

**Figure 2.**
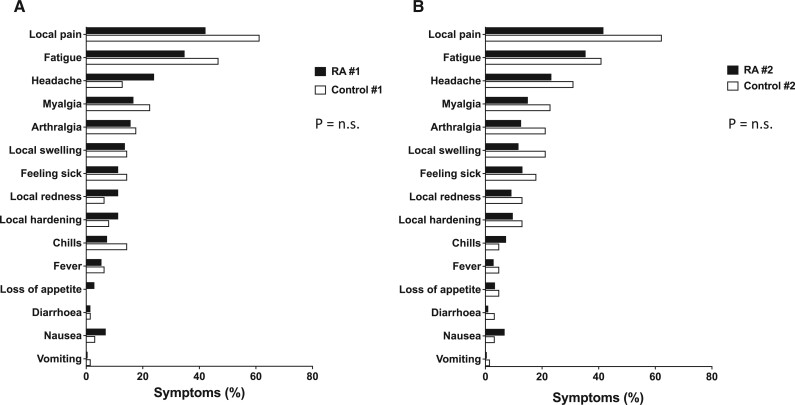
Symptom-based tolerability in the total study sample (*N* = 266). Symptom frequencies are given as percentages on the *x*-axis. Results are stratified for the time of vaccination (panel A: first vaccination; panel B: second vaccination) and for the patient subgroup (RA patients *vs* controls). Symptom distributions did not differ significantly between RA patients and controls

Also on the itemised individual symptom level, no significant differences could be detected between the two patient groups. The frequency distributions of the most common symptoms are therefore presented for the total study sample (*N* = 266) and are shown in Fig. 2A and B, broken down by the factors vaccination time point and patient group: Most frequent and most common physical symptoms associated with vaccination were local pain (injection site), fatigue and headache. Although it was not recorded in a structured way, there was no perceptible increase in unplanned presentations in the form of vaccination reactions or even relapses in the immediate temporal context of vaccination.

**Figure 3.**
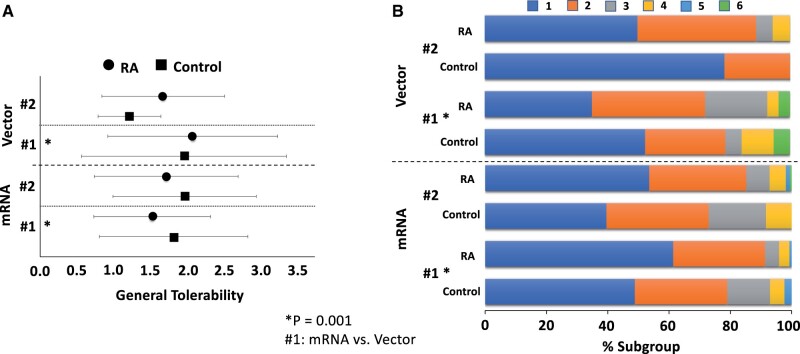
General tolerability by vaccine type (mRNA *vs* vector), patient subgroup (RA patients *vs* controls) and vaccination time point (#1 *vs* #2). Dependent variable (general tolerability; here: *x*-axis) represents a six-point Likert scale (lower = better tolerability). In panel (A), central tendency is represented as group mean ± standard deviation. In panel (B), we present the correspondent data using stacked bar charts for each considered subgroup. The horizontal length of each segment visualizes the percentage belonging to the indicated response category. Simultaneous inclusion of the specified factors in an ordinal regression analysis revealed significantly better tolerability of the mRNA vaccines compared with the vector vaccines for the first vaccination (vacc. #1, mRNA *vs* vector: *P* = 0.001)

However, there was a statistically significant difference in overall self-rated tolerability regarding the first vaccination when comparing subgroups of patients receiving mRNA vaccines (BioNtech, Moderna) *vs* patients with a vector vaccine (AstraZeneca): Ordinal regression analysis revealed that self-rated overall tolerability was significantly better (*P* = 0.001) for the first vaccination in patients with mRNA vaccines (*n* = 193, mean tolerability 1.59) as compared with vector-vaccinated patients (*n* = 73, mean tolerability 2.04; see Fig. 3A and B). In the context of the second vaccination, no significant difference in overall self-rated tolerability was found when comparing the groups with mRNA or vector vaccines (*P* = 0.184).

**Figure 4.**
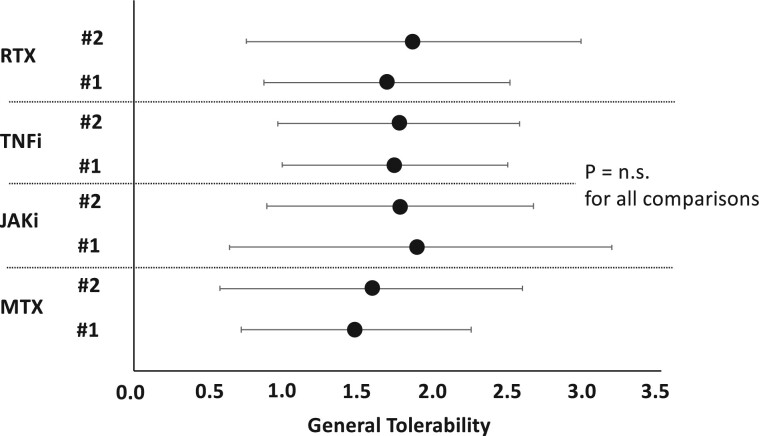
General tolerability within subgroup of RA patients by type of anti-rheumatic medication (MTX *vs* JAKi *vs* TNFi *vs* RTX) and by vaccination time point (#1 *vs* #2). Dependent variable (general tolerability; here: *x*-axis) represents a six-point Likert scale (lower = better tolerability). Central tendency is represented as group mean ± standard deviation. *P* = n.s. for all between-group comparisons

More specifically, we investigated the tolerability of the second vaccination with regard to the possible influence of the factor whether the vaccination scheme was heterologous or homologous. In this context, we were able to show that this independent variable was not significantly associated with self-rated tolerability (*P* = 0.131). Observed means for general tolerability were 1.70 for patients with homologous vaccination (*n* = 243) and 2.0 for study participants with heterologous vaccination (*n* = 23). Furthermore, we found that the type of DMARD used had no significant effect on overall tolerability (Fig. 4), with a trend towards better tolerability in the MTX subgroup.

**Figure 5.**
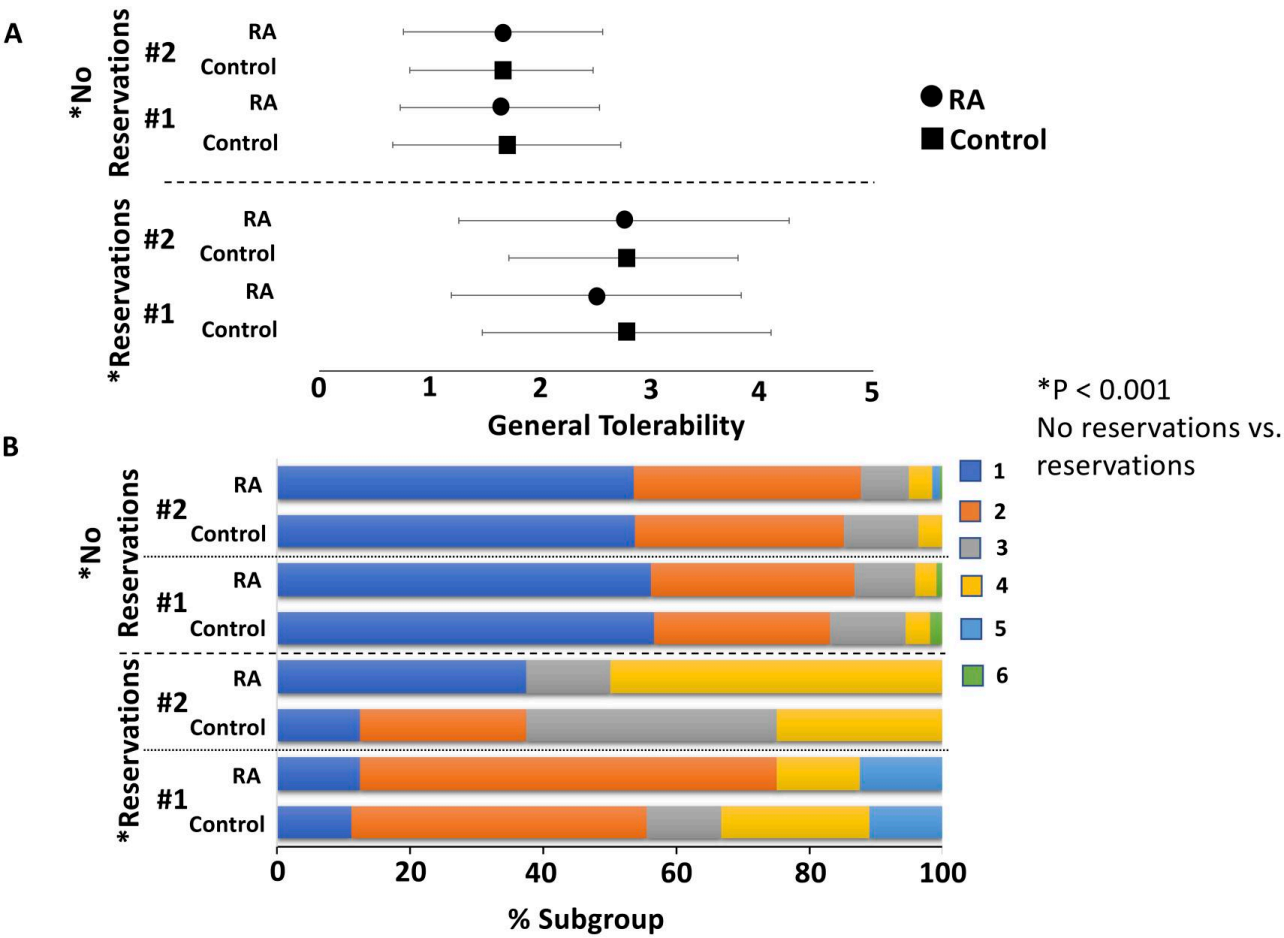
General tolerability by reservations (present *vs* not present), by patient subgroup (RA patients *vs* controls), and by vaccination time point (#1 *vs* #2). Dependent variable (general tolerability; here: *x*-axis) represents a six-point Likert scale (lower = better tolerability). In panel (A), central tendency is represented as group mean ± standard deviation. In panel (B), we present the correspondent data using stacked bar charts for each considered subgroup. The horizontal length of each segment visualizes the percentage belonging to the indicated response category. Simultaneous inclusion of the specified factors in an ordinal regression analysis revealed significantly better tolerability in patients without reservations (*P* < 0.001)

Finally, we assessed the association between subjective reservations and concerns about the COVID-19 vaccines and self-assessed tolerability as well as individual recommendations for future vaccinations: First, it can be generally stated that the majority of all study participants had no reservations about COVID-19 vaccination: 93.6% of individuals expressed no concerns regarding their initial immunization. This value persisted with 94.0% regarding the second vaccination. Particularly, the subgroup of patients with RA even showed significantly fewer concerns about the vaccination. This was true for both the first (96.1% *vs* 85.5%; RA patients *vs* controls; *P* = 0.003) and the second (96.1% *vs* 87.1%; RA patients *vs* controls; *P* = 0.009) vaccination. Although reservations about the vaccination in total were rare (6.4% for the first and 6.0% for the second vaccination), ordinal regression analysis revealed that they significantly predicted poorer overall tolerability (2.65 *vs* 1.65 for the first vaccination and 2.75 *vs* 1.66 for the second vaccination; *P* < 0.001 for both vaccinations; see Fig. 5A and B). In addition, the propensity to recommend future vaccinations was significantly reduced in patients with reservations compared with participants without such concerns with 88.2% *vs* 99.6% for the first vaccination (*P* < 0.001) and 87.5% *vs* 98.4% for the second vaccination (*P* = 0.004).

## Discussion

Despite the general scepticism about vaccination in the context of SARS-CoV-2 that has been widely reported in the media, the results of the presented study on the tolerability of the vaccination from the patient’s perspective are encouraging. Tolerability of vaccination against COVID-19 in our cohort was rated on average on a scale of 1 (very good) to 6 (very poor) as 1.67 for the first vaccination and 1.70 for the second vaccination by patients with RA, and thus, overall, as very good. Tolerability in controls was not significantly different at 1.85 and 1.79 for the first and second vaccination, respectively. The degree of recommendation to other people by those vaccinated was also very high in both patients with RA and controls, exceeding 90% in each case. Our findings are supported largely by data from one of the world’s largest vaccination registries on COVID-19 in the rheumatological patient population, the EULAR Coronavirus Vaccine (COVAX) physician-reported registry. In an evaluation of 5121 participants from 30 countries, 90% of whom had inflammatory rheumatic disease, Machado *et al.* [17] demonstrated that, as in our study, the safety profile and tolerability were comparable between patients with inflammatory rheumatic diseases and those with primarily non-inflammatory joint diseases. The majority of patients in the aforementioned study also tolerated the vaccination very well, relapses of IMIDs were rare overall, and serious adverse events were very rarely reported. The good tolerability of the COVID-19 vaccine in patients with rheumatic diseases has also been reported in other studies [18, 19].

In the safety reports of the Paul-Ehrlich-Institut (PEI), the federal agency responsible for the licencing and monitoring of vaccines in Germany, the three most common adverse reactions associated with COVID-19 vaccination were headache (36 per 100 000 vaccinations), fatigue (31 per 100 000 vaccinations) and pain at the injection site (29 per 100 000 vaccinations) [13]. These were followed by fever (25 per 100 000 vaccinations) and chills (23 per 100 000 vaccinations). In our cohort, local pain at the injection site (46.6%), followed by fatigue (36.8%) and headache (25.2%) were the three most frequently reported adverse reactions and thus, did not differ in quality from those in the general vaccination collective of the PEI. However, the relative frequencies in the PEI registry were considerably lower than in our control group, but also in the group of patients with RA. This may be due to a considerable under-reporting of mainly non-serious adverse reactions to the PEI as an administrative agency. Moreover, there were no significant differences in the tolerability of the different COVID-19 vaccines in the PEI safety database. In our cohort, the tolerability was better only for the first vaccination in favour of the mRNA vaccines with 1.59 compared with the vector vaccine with 2.04 (*P* = 0.004). In contrast, no significant difference was observed for the second vaccination (1.76 *vs* 1.54; *P* = 0.130). Interestingly, we found a trend towards better tolerability when using the same vaccines for the first and second vaccination (homologous vaccination) compared with two different vaccines (heterologous vaccination). A recent paper on patients on dialysis came to a similar conclusion with a slightly worse but still good tolerability of heterologous vaccination compared with a homologous sequence [20]. Due to the small number of cases (*N* = 23) in our cohort and the associated very low statistical power, no statement can be made about differences in tolerability depending on the specific sequence of vaccines used in the case of heterologous vaccination.

The DMARDs used included MTX, JAK inhibitors, TNF inhibitors and RTX. Although there were significant differences in humoral immunogenicity between groups of different DMARD therapies as measured by the level of vaccine titres in our cohort [21, 22], the underlying therapy did not significantly affect the reported tolerability. This is consistent with the results of other studies that have primarily investigated the immunological efficacy of vaccination against COVID-19 in the context of different DMARDs [4, 23–26].

Reservations about COVID-19 vaccination were much less common than expected in our cohort. Only 17 (first vaccination) and 16 (second vaccination) of 266 vaccinated study participants had reservations about COVID-19 vaccination. The proportion of participants with reservations was significantly higher in the control group than among RA patients. According to the authors, this may be due to patients’ awareness of the risk of infection associated with rheumatic disease and DMARD therapy in general. This effect also appears to have offset additional concerns about a potentially reduced efficacy in patients with inflammatory rheumatic diseases in terms of protection against infection or adverse immunological reactions such as triggering of inflammatory relapses [11, 27]. Previously, Nakafero *et al.* [28] did not find a significant association between influenza vaccination and primary care physician consultation based on various markers of increased disease activity or adverse effects of the vaccine in patients with IMIDs (*N* = 14 928). Despite single case reports and small case series, there is also no robust database to date for COVID-19 vaccination that would show a generally increased relapse rate on the part of the underlying rheumatic disease [29, 30]. Furthermore, scientific societies in rheumatology worldwide have published recommendations for vaccination against COVID-19 very early with the availability of the vaccines, always addressing these aspects [7, 11, 31]. Due to the design of our study, which focused on PROs, the main shortcoming in this regard is the lack of data on adverse events including relapses of RA observed by physicians. However, this aspect—on an observational basis—may also be classified as good overall, especially since at the site conducting the study, no increase in unscheduled presentations in terms of vaccination reactions or even relapses was observed. A further limitation of the study is the potential selection bias related to our inclusion criteria: We could not include patients who did not receive a second vaccination due to considerable (e.g. subjective) intolerance of the first vaccination.

Probably the most important finding of our study for routine clinical practice is the strong influence of reservations towards COVID-19 vaccination on the reported tolerability and subsequently also on the degree of further recommendation to others. Reservations were significantly associated with poorer tolerability and with a significantly lower degree of further recommendation. Confidence in the COVID-19 vaccine is a key determinant of vaccine uptake, as recently reaffirmed [32]. This result strongly underlines the importance of pre-existing vaccination scepticism as a predictor of subjectively perceived tolerability and attitudes towards subsequent vaccinations, independent of actual experience. This aspect also emphasizes the need to continue to objectively and systematically record adverse events related to vaccination in the future and to continuously assess the safety of vaccinations. Thus, potential safety signals can be identified in a timely manner, and confidence in vaccinations can further be improved.

## Supplementary Material

rkae111_Supplementary_Data

## Data Availability

The data underlying this article will be shared on reasonable request to the corresponding author.
