# Supplementary material for: Real-world data on tolerability of COVID-19 vaccination in patients with rheumatoid arthritis based on patient-reported outcomes
Source: Rheumatol Adv Pract. 2024 Sep 5;8(4):rkae111. doi: 10.1093/rap/rkae111 (PMC11398971; doi:10.1093/rap/rkae111)
Supplement: rkae111_Supplementary_Data [file rkae111_supplementary_data.zip › 24-032 Supplementary Data S1 - Questionnaire.pdf]

Appendix: SARS-CoV-2 Vaccination Tolerability Questionnaire

Please indicate whether you were aware of an infection with the coronavirus (SARS-CoV-2) at any point prior to receiving the first and second vaccination.

□ Yes □ No

Please respond to the following questions regarding the **first vaccination against SARS-CoV-2**.

Date of 1st vaccination [dd.mm.yyyy]: ______. _______. 20 ______

Type of vaccine [manufacturer]:

□ BIONTECH □ Moderna

□ AstraZeneca □ other than the above mentioned:_______________

Please indicate whether you have experienced any of the following adverse effects:

	no	yes		no	yes
Local pain			Diarrhoea		
Local redness			Headache		
Local swelling			Fatigue		
Local hardening			Myalgia		
Nausea			Arthralgia		
Vomiting			Fever (>38,5°C)		
Loss of appetite			Chills		
Feeling sick			Unable to work		
Medical treatment required due to a vaccine reaction					

How would you rate your overall tolerability (school grades 1-6)?

□ 1 (very good)

□ 2

□ 3

□ 4

□ 5

□ 6 (very bad)

Based on your experience, would you recommend the vaccination to others?

□ Yes

□ No

Has your personal experience changed your attitude towards vaccination?

□ Yes

□ No

Please respond to the following questions regarding the **second vaccination against SARS-CoV-2**.

Date of 2nd vaccination [dd.mm.yyyy]: ______. _______. 20 ______

Type of vaccine [manufacturer]:

□ BIONTECH □ Moderna

□ AstraZeneca □ other than the above mentioned:_______________

Please indicate whether you have experienced any of the following adverse effects:

	no	yes		no	yes
Local pain			Diarrhoea		
Local redness			Headache		
Local swelling			Fatigue		
Local hardening			Myalgia		
Nausea			Arthralgia		
Vomiting			Fever (>38,5°C)		
Loss of appetite			Chills		
Feeling sick			Unable to work		
Medical treatment required due to a vaccine reaction					

How would you rate your overall tolerability (school grades 1-6) in the context of the second vaccination?

□ 1 (very good)

□ 2

□ 3

□ 4

□ 5

□ 6 (very bad)

Based on your experience, would you recommend the vaccination to others?

□ Yes

□ No

Has your personal experience changed your attitude towards vaccination?

□ Yes

□ No
