# Supplementary material for: Real-world data on tolerability of COVID-19 vaccination in patients with rheumatoid arthritis based on patient-reported outcomes
Source: Rheumatol Adv Pract. 2024 Sep 5;8(4):rkae111. doi: 10.1093/rap/rkae111 (PMC11398971; doi:10.1093/rap/rkae111)
Supplement: rkae111_Supplementary_Data [file rkae111_supplementary_data.zip › 24-032 Supplementary Figures.docx]

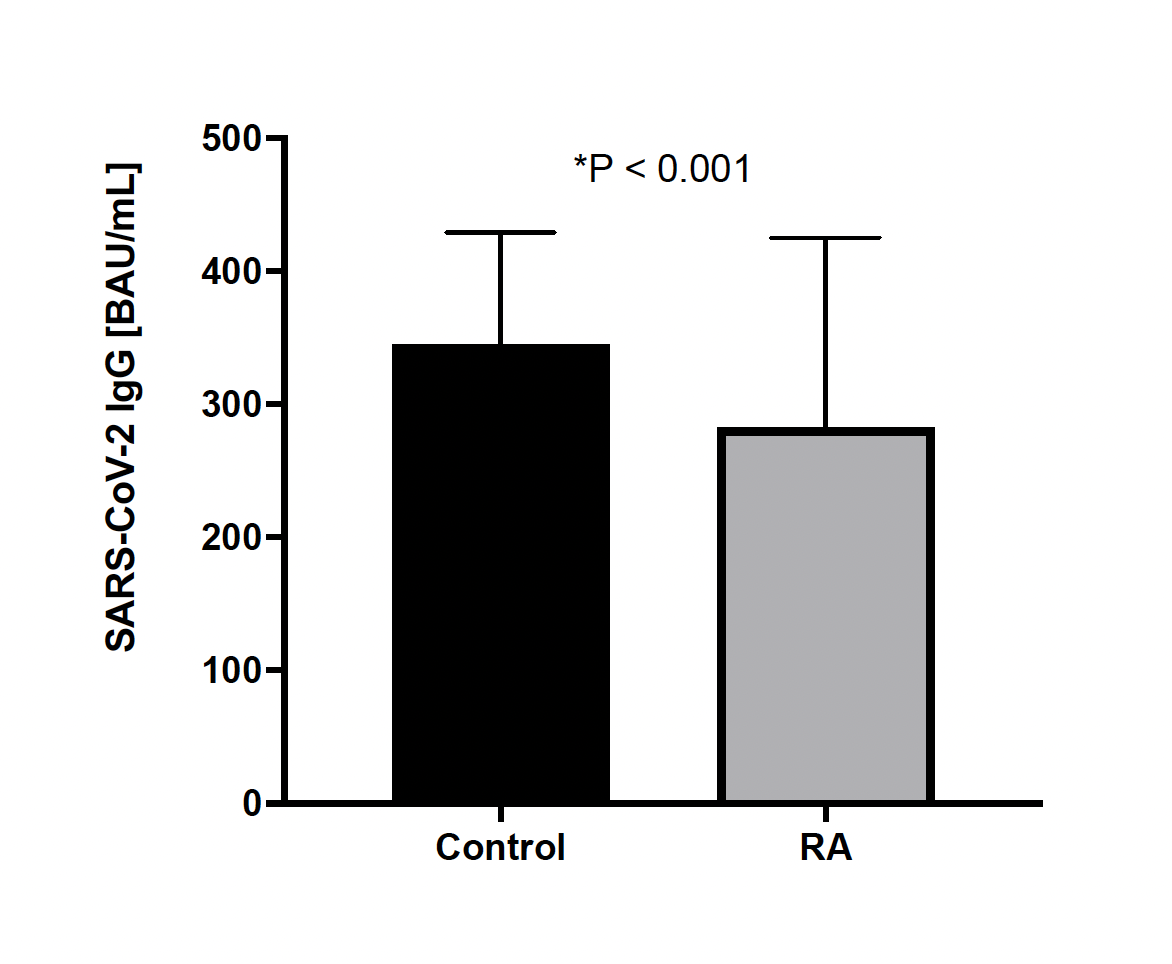


**Supplementary Figure S1**. **Humoral immune response by patient subgroup (control vs. RA patients).** Dependent variable represents SARS-CoV-2 IgG antibody titres (BAU per mL). Central tendency is represented as group mean ± standard deviation. Immune response is significantly weaker in RA patients (P <0.001).


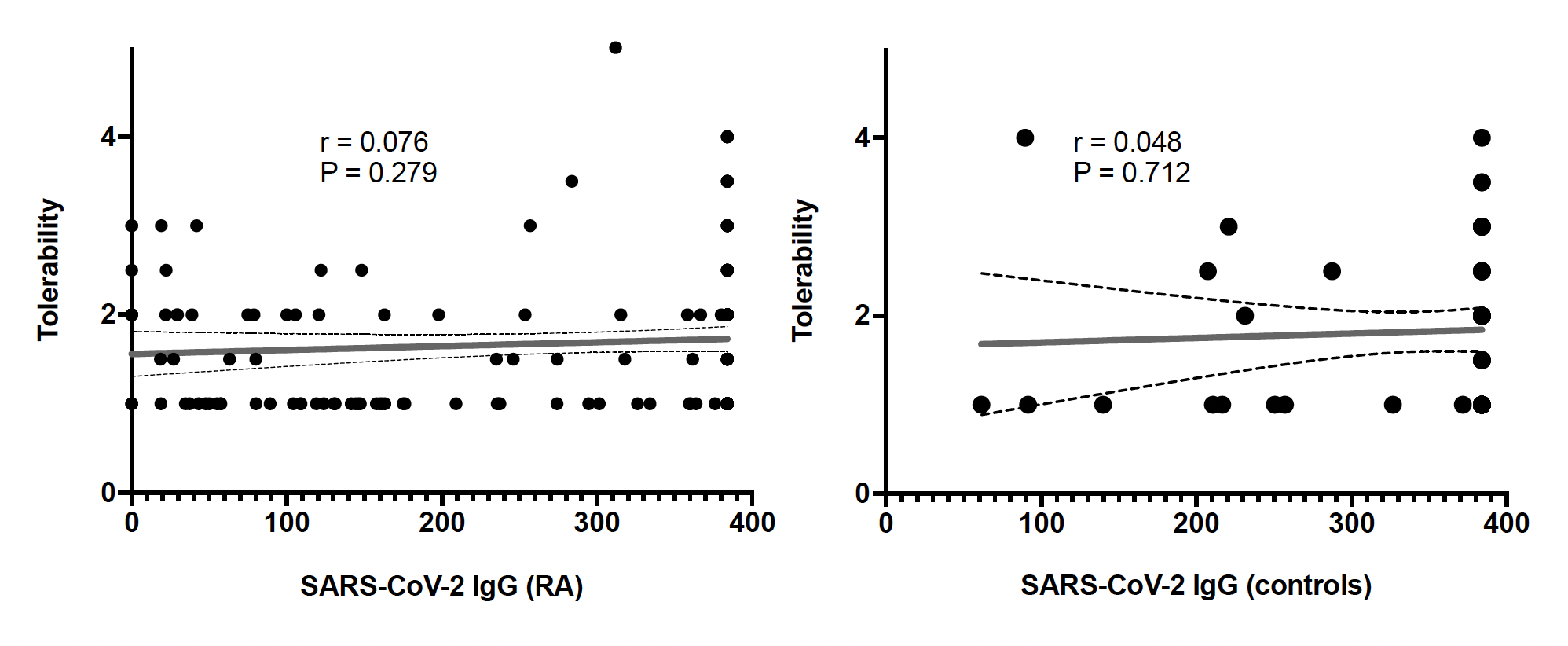


**Supplementary Figure S2**. **Bivariate correlation between self-reported vaccination tolerability (y-axis; 6-point Likert scale, lower is better) and humoral immune response (x-axis; SARS-CoV-2 IgG [BAU per mL]).** Scatter plots with regression lines and the corresponding 95% confidence intervals are displayed separately for both subgroups (left panel: RA; right panel: controls). For both subgroups, there is no significant linear relationship between tolerability and immune response: The magnitude of the correlation coefficient is close to zero for each subgroup, the coefficients are not statistically significant, and there is a relatively wide 95% confidence interval, indicating the absence of a relevant linear association.
